# A horizontally acquired expansin gene increases virulence of the emerging plant pathogen *Erwinia tracheiphila*

**DOI:** 10.1038/s41598-020-78157-w

**Published:** 2020-12-10

**Authors:** Jorge Rocha, Lori R. Shapiro, Roberto Kolter

**Affiliations:** 1 Department of Microbiology, Harvard Medical School, Boston, MA USA; 2 Present Address: Conacyt-Centro de Investigación y Desarrollo en Agrobiotecnología Alimentaria, San Agustin Tlaxiaca, 42163 Hidalgo Mexico

**Keywords:** Ecology, Evolution, Microbiology, Molecular biology

## Abstract

*Erwinia tracheiphila* is a bacterial plant pathogen that causes a fatal wilt infection in some cucurbit crop plants. Wilt symptoms are thought to be caused by systemic bacterial colonization through xylem that impedes sap flow. However, the genetic determinants of within-plant movement are unknown for this pathogen species. Here, we find that *E. tracheiphila* has horizontally acquired an operon with a microbial expansin (*exlx*) gene adjacent to a glycoside hydrolase family 5 (*gh5*) gene. Plant inoculation experiments with deletion mutants in the individual genes (Δ*exlx* and Δ*gh5*) and the full operon (Δ*exlx*–*gh5*) resulted in decreased severity of wilt symptoms, decreased mortality rate, and impaired systemic colonization compared to the Wt strain. Co-inoculation experiments with Wt and Δ*exlx*–*gh5* rescued the movement defect of the mutant strain, suggesting that expansin and GH5 function extracellularly. Together, these results show that expansin–GH5 contributes to systemic movement through xylem, leading to rapid wilt symptom development and higher rates of plant death. The presence of expansin genes in diverse species of bacterial and fungal wilt-inducing pathogens suggests that microbial expansin proteins may be an under-appreciated virulence factor for many pathogen species.

## Introduction

The surfaces of all land plants are colonized by complex microbial communities. For a microbe, the ability to colonize a plant increases access to the nutritional resources produced by that plant^1,2^. This has driven the evolution of diverse molecular mechanisms for plant colonization throughout commensal, beneficial and pathogenic microbes^3^. Proteins called ‘expansins’ are particularly intriguing, and genes coding for expansins are being identified in the genomes of an increasing number of plant-associated bacterial and fungal species^4–7^. Expansins are non-enzymatic, two-domain proteins of ~ 250 amino acids. The expansin N-terminal domain is related to glycoside hydrolase family 45 functional domains and the C-terminal domain is related to grass pollen allergens^8–10^. In all species of land plants and green algae, expansin-coding genes are ubiquitous and fulfill the essential role of non-enzymatically loosening cell wall cellulose and enabling cell wall extension during normal growth^8,11–15^.

Expansin-encoding genes have also been identified in hundreds of taxonomically diverse bacterial and fungal species which do not have cellulosic cell walls, but interact with live or dead plant or algal matter^4,6,7,16^. In these microbial species, expansin proteins are hypothesized to promote colonization of plants through interactions with structural cellulose and/or hemicellulose in plant cell walls^4,7,17^. However, the functions and importance of microbial expansins for plant colonization have been empirically investigated in very few microbial species^4^. For several microbes that are non-pathogenic, expansins increase colonization efficiency of plant surfaces^4,17–19^. The microbial expansin from the plant commensal bacterium *Bacillus subtilis* (BsEXLX1) has only a fraction of the cellulose loosening activity against plant cell walls in vitro compared to plant expansins. Yet, BsEXLX1 deletion mutants are either severely impaired or unable to successfully colonize the surface of maize roots^17,20^. In some species of plant beneficial fungi, expansins (also referred to as ‘swollenins’) increase fungal mutualistic capabilities towards plant hosts^18,19^. Expansin function has also been investigated in several species of plant pathogens. In the bacterial plant pathogen *Ralstonia solanacearum,* an expansin deletion mutant has decreased virulence^21^. Expansin from *Pectobacterium atrosepticum* contributes to virulence in potato, and induces plant defense responses^22^. For *Clavibacter michiganensis*, studies have described contradictory expansin roles for virulence and ability to colonize xylem^21,23–26^. Outside of these few examples, fundamental questions surrounding how microbial expansins mediate plant colonization, and the molecular mechanism(s) by which these proteins interact with plant structural carbohydrates remain unknown for almost all species^4,6,7,16,17,21,24,27,28^.

**Figure 1:**
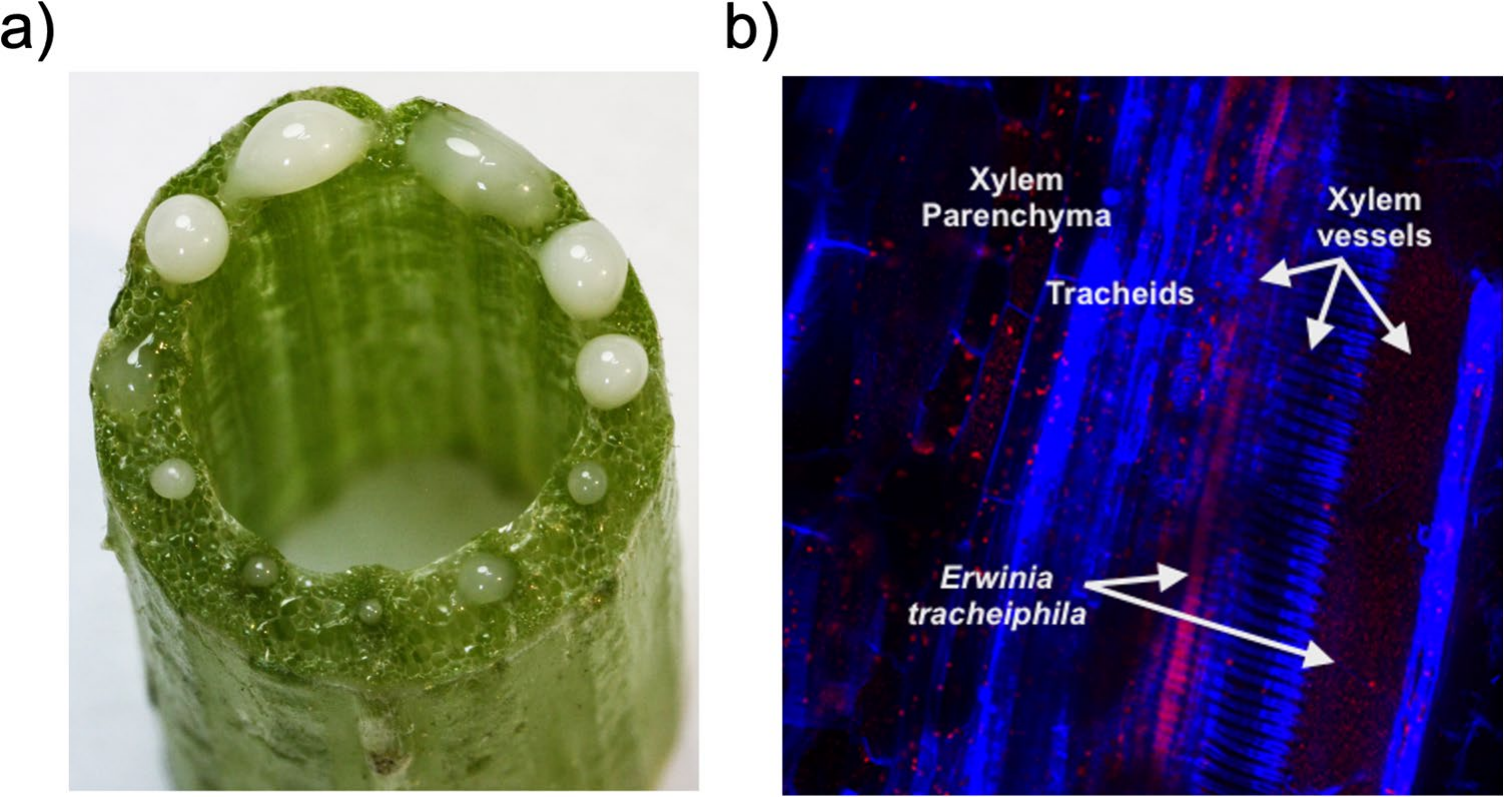
Xylem colonization by *E. tracheiphila*. (**a**) Visible strain of *E. tracheiphila* (Wt) oozing from xylem in all vascular bundles of a symptomatic plant after a horizontal stem cross section cut. (**b**) ×20 confocal microscopy image of a longitudinal section of a symptomatic, *E. tracheiphila* infected *Cucurbita pepo* stem. Image is falsely colored so that plant structures are shown in blue and live *E. tracheiphila* bacterial cells are red.

The bacterial plant pathogen *Erwinia tracheiphila* Smith (Enterobacteriaceae)*,* the causative agent of bacterial wilt of cucurbits, contains an operon with an expansin gene (*exlx*) and a glycoside hydrolase family 5 gene (*gh5*)^7,29–31^. *E. tracheiphila* colonizes host plant xylem and causes a fatal bacterial wilt infection in only two genera of cucurbit host plants: cultivars of *Cucurbita* spp. (summer and winter squash) and *Cucumis* spp. (cucumber and muskmelon)^32^. The *E. tracheiphila* genome has undergone dramatic structural changes consistent with an evolutionarily recent emergence into a novel host plant population, including the horizontal acquisition of multiple genes likely important for virulence^32–36^. Unlike most bacterial plant pathogens, *E. tracheiphila* cannot persist in environmental reservoirs^34,37,38^. Instead, *E. tracheiphila* is obligately transmitted by two species of highly specialized leaf beetle vectors^39–42^. Pathogen transmission can occur when *E. tracheiphila* cells in frass from infective beetles is deposited near recent foliar feeding wounds or on floral nectaries^40,43^. Bacteria can then move systemically through xylem and block sap flow to induce systemic wilting (Fig. 1), which is followed by plant death within 2–3 weeks after the first wilt symptoms appear^33,44–46^. *E. tracheiphila* costs cucurbit farmers millions of dollars annually through direct yield losses and the expense of indirect control measures^32^. Despite the economic burden caused by *E. tracheiphila*, no genetic determinants of bacterial pathogenesis or virulence have yet been empirically determined.

Here, we reconstruct the evolutionary histories of both the *exlx* and *gh5* open reading frames (ORFs) in *E. tracheiphila*, and characterize the role of expansin–GH5 for colonization of squash (*Cucurbita pepo*) host plants. We find that the phylogenies of both *exlx* and *gh5* are consistent with horizontal acquisition by *E. tracheiphila*. In planta inoculation experiments with deletion mutants for *exlx* and *gh5* show these genes significantly increase the ability of *E. tracheiphila* to systemically colonize xylem (Fig. 1), induce wilt symptoms, and cause high rates of plant death. Co-inoculation experiments with Wt and deletion mutants suggest that these proteins likely function extracellularly, probably as an assembled EXLX–GH5 complex. Together, these results suggest that the *Et–exlx–gh5* locus is a non-canonical yet potent virulence factor, and horizontal acquisition of this locus was a key event driving the recent emergence of *E. tracheiphila*^32,35^ as a virulent plant pathogen that causes high mortality by inducing wilt symptoms and plant death by blocking the flow of xylem sap (Fig. 1). These findings highlight the continued risk of horizontal gene transfer driving an increase in pathogen virulence, and the continuing vulnerability of agricultural populations to invasion by virulent new pathogens.

## Results

### Identification of a locus with an expansin gene in *Erwinia tracheiphila*

**Figure 2:**
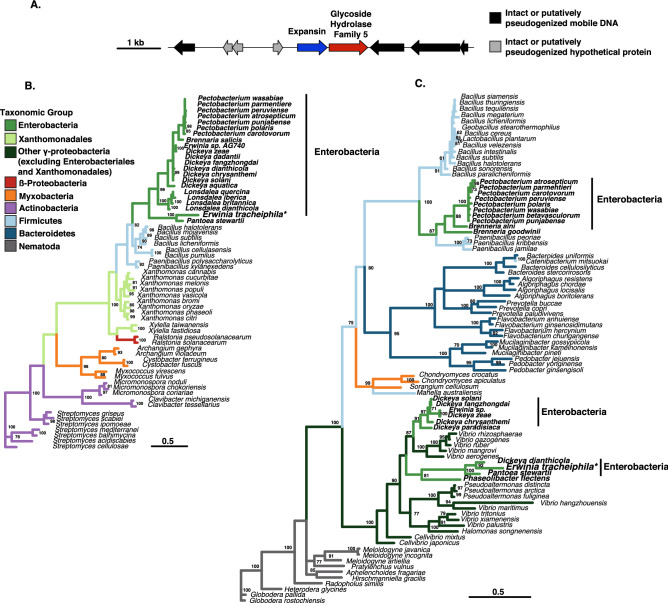
Genomic context and phylogenies of the expansin and glycoside hydrolase 5 genes in *Erwinia tracheiphila. *(**A**) Genomic context of the expansin (*exlx*) and glycoside hydrolase 5 (*gh5*) open reading frames (ORFs) in *Erwinia tracheiphila.* The ORFs and intergenic spaces are drawn to scale, with the black line representing position on the chromosome, and each ORF as an arrow color-coded according to ab initio annotated function. The scale bar is the length in nucleotides of the ORFs and intergenic spaces. (**B**) Distribution of expansin (*exlx*) homologs in a taxonomically representative set of bacterial species. Branches are colored according to taxonomic assignments. The tree was reconstructed using maximum likelihood and should be considered unrooted. Numbers at nodes are bootstrap pseudoreplicates, and the scale bar is the number of amino acid substitutions per site. (**C**) Distribution of glycoside hydrolase 5 (*gh5*) homologs in a taxonomically representative set of species. Branches are colored according to taxonomic assignments, using the same color assignments as Fig. 1b. The tree was reconstructed using maximum likelihood and should be considered unrooted. Numbers at nodes are bootstrap pseudoreplicates, and the scale bar is the number of amino acid substitutions per site.

We identified a locus with two open reading frames (ORFs) flanked by mobile DNA elements during manual curation of ab initio gene predictions in the *E. tracheiphila* reference strains (Fig. 2A)^29,30^. The first ORF, *Et*–*exlx* (AXF78871.1)*,* is predicted to encode a protein product with 243 amino acids containing both domains found in canonical expansin proteins^14,47^. The second ORF, *Et*–*gh5* (AXF77819.1)*,* has 315 codons and is predicted to encode a putatively pseudogenized endo-1,4-beta-xylanase A precursor (EC 3.2.1.8) with a glycoside hydrolase family 5 (GH5) functional domain (www.CAZy.org)^48^. Many *Et*–*gh*5 homologs in the NCBI *nr* database contain 415–450 amino acids, and RAST ab initio gene annotation predicts that the truncation to 315 amino acids eliminates cellulase activity and renders *Et–gh5* non-enzymatic^49^. The sequences of both ORFs predict a signal peptide for secretion and a Signal Peptidase cleavage site^50^, suggesting that each protein product is individually secreted.

### The phylogenies of expansin and GH5 are consistent with horizontal gene transfer

In *E. tracheiphila,* the *exlx* and *gh5* ORFs are flanked by mobile DNA elements (Fig. 2A). Because mobile DNA are common agents of horizontal gene transfer^35,51,52^, we reconstructed the phylogenies of both the *exlx* and *gh5* genes and compared the phylogenies of these two genes to the bacterial species phylogeny^7,35,51–57^. We find that the phylogenies of both *Et*–*exlx* and *Et*–*gh5* genes are in conflict with the species phylogeny, which is consistent with horizontal acquisition of these genes by *E. tracheiphila* (Fig. 2B,C). *E. tracheiphila* and *Pantoea stewartii* are the only species with microbial expansin homologs from the *Erwinia* and *Pantoea* genera, respectively (Fig. 2B). The *E. tracheiphila* and *P. stewartii* expansin homologs group with the other Enterobacterial plant pathogens (*Pectobacterium* spp. and *Dickeya* spp.), suggesting horizontal transfer has occurred between these species^58^.

The bacterial expansin phylogeny is consistent with additional horizontal gene transfer events, which is in agreement with previous studies^6,7^. There is a second group of γ-proteobacterial expansin homologs in Xanthomonadaceae, and these two distinct groups of γ-proteobacterial *exlx* homologs are separated by a group of Firmicutes *exlx* homologs^7^. The phylogenetic reconstruction is also consistent with an expansin acquisition by the β-proteobacterial plant pathogen *Ralstonia solancearum* from a Xanthomonadaceae donor (Fig. 2B)^6,7^.

The *Et*–*gh5* phylogeny is also consistent with multiple horizontal gene transfer events (Fig. 2C). Overall, *Et*–*gh5* homologs have a relatively sparse distribution in some Enterobacteriaceae, Firmicutes, Myxobacteria, Bacteroidetes and β-proteobacteria, and in some species of plant pathogenic nematodes^57^. In Enterobacteriaceae, the *gh5* homologs separate into three distinct groups. One group is comprised of *E. tracheiphila, Pantoea stewartii, Dickeya dianthicola,* and *Phaseolibacter* sp. (recently reclassified to Enterobacteriaceae^59^)*.* The *gh5* homologs from the other plant-pathogenic *Dickeya* spp. comprise a second group of Enterobacterial *gh5* homologs, and plant-pathogenic *Pectobacterium* and *Brennaria* spp. are a third group (Fig. 2C). These three groups of Enterobacterial *gh5* homologs are dispersed among the *gh5* homologs from Firmicutes, Bacteroidetes, Myxobacteria and other lineages of γ-proteobacteria*,* which is consistent with multiple HGT events between these bacterial lineages.

### Co-occurrence patterns of expansin and glycoside hydrolase 5 genes in bacteria

**Figure 3:**
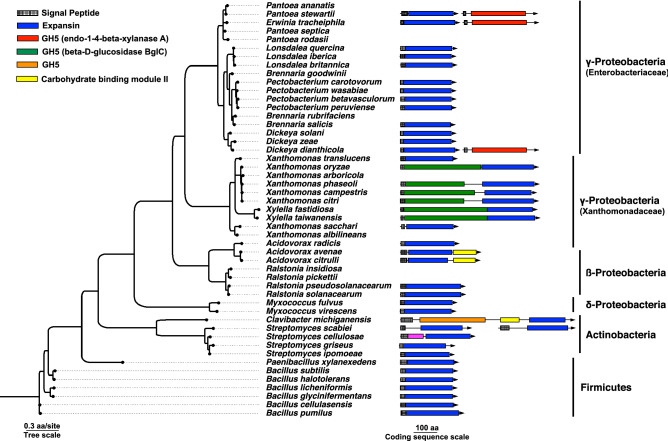
Co-occurrence of expansin genes with carbohydrate active domains. The *gyrB* species tree of selected bacteria with an expansin gene, and several species without expansins. The expansin and carbohydrate active domains are depicted as arrows. The rectangles within the arrows indicate whether that ORF has an expansin domain, a carbohydrate active domain, or both. Homologous carbohydrate active domains are color-coded. Both expansin genes are shown for *Streptomyces scabiei,* the only microbial species to harbor two expansin homologs with signal peptides for secretion in one genome. Accession numbers of the depicted protein sequences, and accession numbers of several expansin homologs that do not have predicted signal peptides for secretion and are not depicted in the figure, can be found in https://github.com/lshapiro31/gh5.expansin.phylogenetics. The domains are drawn to scale. The tree was reconstructed using maximum likelihood with 100 bootstrap pseudoreplicates and should be considered unrooted.

In bacteria, the *exlx* gene can be found in species that (1) do not harbor a *gh5* anywhere in their genomes, (2) harbor a *gh5* ORF in the same genome but physically distant from *exlx,* or (3) encode a *gh5* adjacent to an *exlx* in the same locus (Fig. 3). A *gh5* homolog is present in the genomes of multiple plant-pathogenic *Pectobacterium* and *Dickeya* species that harbor an *exlx* homolog, but these ORFs do not co-occur in the same operon (Fig. 2B,C). Only three enterobacterial species (*E. tracheiphila, Pantoea stewartii,* and *Dickeya dianthicola*) harbor *exlx* and *gh5* as two distinct ORFs that co-occur in the same operon. In these three species, the *exlx* and *gh5* genes have distinct signal peptides and are separated by ~ 50 nucleotides. In *P. stewartii,* the *exlx–gh5* locus is on a plasmid (pDSJ08)*,* which may increase the probability of acting as a donor for horizontal gene transfer.

A *gh5* is fused to an *exlx* as a single ORF in many plant pathogenic Xanthomonadaceae^7,60^. This *gh5* domain in Xanthomonadaceae is non-homologous to the GH5 domain in Enterobacteriaceae, and the *exlx* and *gh5* domain structure in *E. tracheiphila*, *P. stewartii* and *D. dianthicola* is in reverse orientation compared to the *gh5*–*exlx* domain order in Xanthomonadaceae. A distinct *gh5* domain truncated to 289 amino acids is found in *Clavibacter michiganensis* (*CelA*)*,* and this is the only known microbial expansin that is fused to both a GH5 and CBM2 domain in a single coding sequence^25^. Some isolates of *C. michiganensis* contain an additional *exlx* ORF that is not fused to either a GH5 or CBM2 domain^6,16,21^. The three non-homologous *gh5* domains that are adjacent or fused to *exlx* genes in distinct bacterial lineages and with distinct domain architectures is consistent with at least three independent fusion events, and may be an example of functional convergence.

### Expansin and GH5 genes both contribute to *Erwinia tracheiphila* virulence

To evaluate the individual and combined roles of expansin and GH5 for *E. tracheiphila* virulence, as well as the possible synergistic effects of both proteins, we generated a deletion mutant of the complete operon (strain Δ*exlx–gh5*), as well as single-ORF mutants in only the expansin ORF (strain Δ*exlx*) and only the *gh5* ORF (strain Δ*gh5*). Strains that complemented the three deletion mutations (Δ*exlx–gh5*(cEXLX-GH5), Δ*exlx*(cEXLX) and Δ*gh5*(cEXLX-GH5), respectively) were also constructed (Supplemental Table S1). Variation in virulence was quantified via differences in days until (1) development of wilt symptoms localized to the inoculated leaf, (2) systemic spread of wilt symptoms to a second non-inoculated leaf, and (3) plant death.

**Figure 4:**
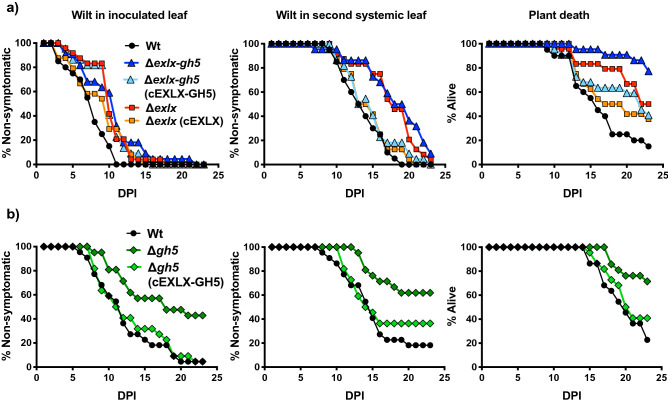
Contribution of the *Erwinia tracheiphila exlx–gh5* locus to wilt symptom development and plant death. (**a**) In planta inoculation experiment comparing virulence of Wt, Δ*exlx–gh5,* Δ*exlx–gh5*(cEXLX-GH5), Δ*exlx* and Δ*exlx*(cEXLX) strains. (**b**) A second independent in planta inoculation experiment comparing virulence of Wt, Δ*gh5,* and Δ*gh5*(cEXLX-GH5) strains. In both (**a**) and (**b**), inoculated plants were monitored for first appearance of wilt symptoms in the inoculated leaf, first appearance of systemic wilt symptoms in a second non-inoculated leaf and plant death for 23 days post inoculation (DPI). Summary statistics are in Table 1 and statistical analyses are in Table 2.

**Figure 5:**
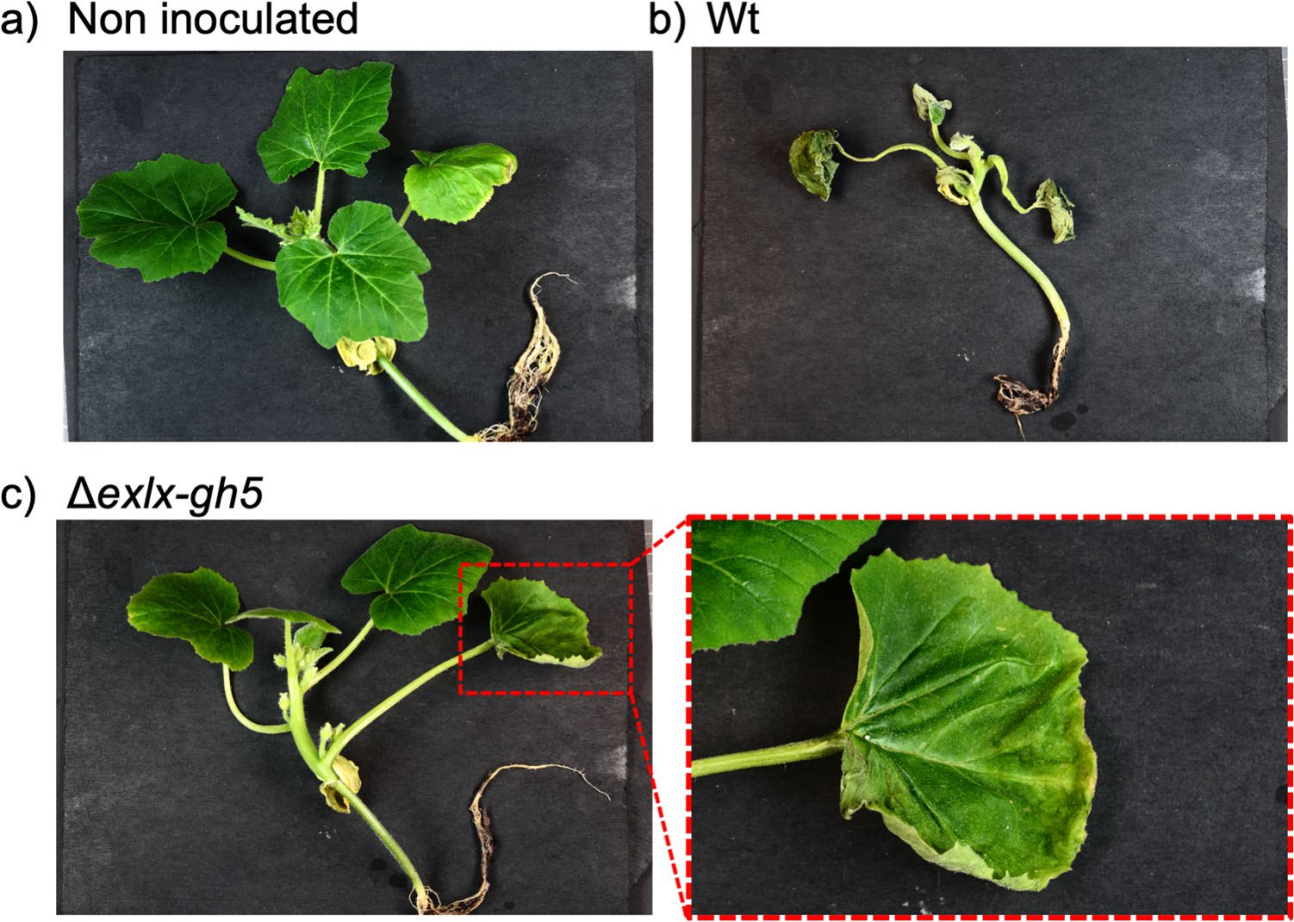
Visual comparison of wilt symptoms in squash seedlings after inoculation with either the Δ*exlx–gh5* mutant or wild type *Erwinia tracheiphila*. (**a**) Non-inoculated squash seedling with no wilt symptoms (**b**) Squash seedling inoculated with wild type *E. tracheiphila* that has developed systemic wilt symptoms (**c**) Representative symptoms caused by inoculation with the Δ*exlx–gh5* mutant strain, where wilt often remains localized to the inoculated leaf without causing systemic wilt symptoms.

Plants inoculated with Wt suffered high mortality (85%; 17 out of 20 plants), but significantly fewer plants inoculated with Δ*exlx–gh5* died (22%; 5 of 22) (Fig. 4a, Table 1). Plants inoculated with Δ*exlx*–*gh5* had delayed development of initial wilt symptoms in the inoculated leaf, and delayed appearance of systemic wilt symptoms in a non-inoculated leaf compared to plants inoculated with Wt (Fig. 4a, Tables 1 and 2). Further, wilt symptoms in plants inoculated with Δ*exlx–gh5* were more likely to be localized to the inoculated leaf (i.e., symptoms did not progress to systemic infection or plant death) compared to plants inoculated with Wt (Fig. 5). The complemented strain Δ*exlx–gh5*(cEXLX-GH5) had restored ability to induce wilting symptoms at a second non-inoculated leaf, and partially restored the mortality rate (59%; 13 out of 22; Fig. 4a, Tables 1 and 2).

**Table 1:** Summary of in planta inoculation experiments comparing virulence between strains Wt, Δ*exlx–gh5*, Δ*exlx–gh5*(cEXLX-GH5), Δ*exlx* and Δ*exlx* (cEXLX) (Fig. 4a); and between strains Wt, Δ*gh5* and Δ*gh5*(cEXLX-GH5) (Fig. 4b).

Strain	Average number of days until			Number of plants with symptoms at end of experiment (day 23)			Total plants
	Local wilt symptoms (first leaf)	Systemic wilt symptoms (second leaf)	Death	Local wilt symptoms (first leaf)	Systemic wilt symptoms (second leaf)	Died	
**Figure ****4****a. Wt, Δexlx–gh5, Δexlx–gh5(cEXLX-GH5), Δexlx and Δexlx(cEXLX)**							
Wt	7.4	13.7	15.29	20 (100%)	20 (100%)	17 (85%)	20
Δ*exlx–gh5*	10.45	17.65	19.4	22 (100%)	20 (91%)	5 (22%)	22
Δ*exlx–gh5*(cEXLX-GH5)	10.14	14.77	16.31	22 (100%)	22 (100%)	13 (59%)	22
Δ*exlx*	10.29	17.43	20.33	24 (100%)	23 (95%)	12 (50%)	24
Δ*exlx*(cEXLX)	8.83	14.46	15.53	24 (100%)	24 (100%)	15 (62%)	24
**Figure ****4****b. Wt, Δgh5 and Δgh5(cEXLX-GH5)**							
Wt	11.82	13.61	18.82	22 (100%)	18 (81%)	17 (77%)	22
Δ*gh5*	13.25	15.38	19.33	12 (57%)	8 (38%)	6 (28%)	21
Δ*gh5*(cEXLX-GH5)	12.67	13	18.54	21 (95%)	14 (66%)	13 (59%)	22

**Table 2:** Results of log-rank Mantel-Cox tests for assessing statistical differences in virulence experiment comparing strains Wt, Δ*exlx–gh5*, Δ*exlx–gh5*(cEXLX-GH5), Δ*exlx* and Δ*exlx*(cEXLX) (Fig. 4a); and comparing strains Wt, Δ*gh5* and Δ*gh5*(cEXLX-GH5) (Fig. 4b).

Compared treatment groups	First leaf symptoms		Second systemic leaf symptoms		Death of plants	
	χ^2^	*P* value	χ^2^	*P* value	χ^2^	*P* value
**Figure ****4****a. Wt, Δexlx–gh5, Δexlx–gh5(cEXLX-GH5), Δexlx and Δexlx(cEXLX)**						
All groups	17.45	0.0016***	28.51	< 0.0001***	22.02	0.0002***
Wt vs. Δ*exlx-gh5*	9.787	0.0018***	16.4	< 0.0001***	20.87	< 0.0001***
Wt vs. Δ*exlx-gh5*(cEXLX-GH5)	11.04	0.0009***	1.5	0.2206	3.939	0.0472*
Δ*exlx-gh5* vs. Δ*exlx-gh5*(cEXLX-GH5)	0.5287	0.4672	7.931	0.0049***	6.841	0.0089**
Wt vs. Δ*exlx*	12.09	0.0005***	16.44	< 0.0001***	9.348	0.0022*
Wt vs. Δ*exlx*(cEXLX)	4.321	0.0376*	0.9923	0.3192	2.497	0.1141
Δ*exlx* vs. Δ*exlx*(cEXLX)	1.014	0.3139	10.19	0.0014***	1.467	0.2258
**Figure ****4****b. Wt, Δgh5 and Δgh5(cEXLX-GH5)**						
All groups	15.53	0.0004***	9.216	0.01*	9.833	0.0073***
Wt vs. Δ*gh5*	14.11	0.0002***	9.906	0.0016***	10.25	0.0014***
Wt vs. Δ*gh5*(cEXLX-GH5)	1.059	0.3034	0.8276	0.363	1.187	0.276
Δ*gh5* vs. Δ*gh5*(cEXLX-GH5)	8.578	0.0034***	4.445	0.035*	4.134	0.042*

*Significant at *P* < 0.05.

**Significant at *P* < 0.01.

***Significant at *P* < 0.005.

Individual deletions of the *Et–exlx* and *Et–gh5* ORFs also caused a decrease in virulence and mortality compared to Wt (Fig. 4, Tables 1 and 2). Plants inoculated with either Δ*exlx* or Δ*gh5* exhibited significant delays in the initial appearance of wilt symptoms in the inoculated leaf, delays in the appearance of systemic wilt symptoms in a second leaf and reduced mortality compared to Wt (Fig. 4, Tables 1 and 2). Genetic complementation of Δ*exlx* (strain Δ*exlx*(cEXLX)) caused partial recovery of virulence by restoring the ability to cause systemic wilt symptoms and plant death (Fig. 4a, Tables 1 and 2). Complementation of Δ*gh5* (strain Δ*exlx–gh5*(EXLX–GH5)) restored the Wt ability to cause wilt symptoms in the inoculated leaf, systemic wilt symptoms in a second leaf, and plant death (Fig. 4b, Tables 1 and 2).

### The *Δexlx–gh5* mutant is impaired in systemic movement

The correlation between within-plant movement of *E. tracheiphila* to systemic wilt symptom development and plant death has been hypothesized, but not yet demonstrated^32,61^. It is assumed that systemic movement of bacteria through xylem—along with bacterial replication far from the initial inoculation point—is necessary to occlude xylem vessels to cause wilt symptoms and plant death (Fig. 1)^32,61^. Both the *Δexlx* and *Δgh5* single mutants and the *Δexlx–gh5* double mutant caused a delay in systemic symptom development (Fig. 4), suggesting both *Et–exlx* and *Et–gh5* ORFs are needed for normal within-plant movement. Because both ORFs appear to be needed for Wt wilt symptom development, we compared the Δ*exlx–gh5* to the Wt strain to test how severity of wilt symptoms correlates with within-plant colonization.

**Figure 6:**
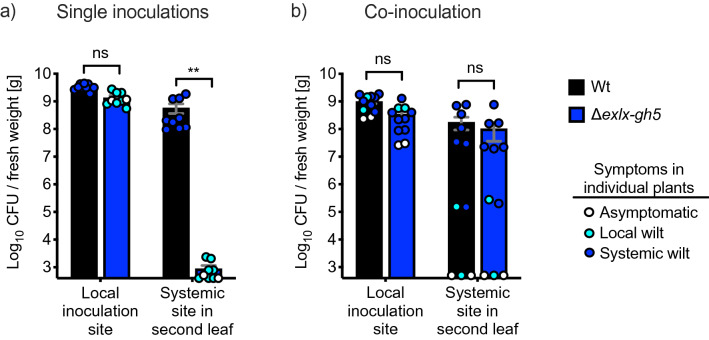
Colonization of Wt and Δ*exlx–gh5* strains in plant xylem. (**a**) Squash seedlings were inoculated with either Wt or Δ*exlx–gh5.* At 12 days post inoculation (DPI), bacterial concentration was determined in the inoculation site and in a petiole of a second, non-inoculated leaf. (Sample sizes, n = 9 per treatment). (**b**) In trans complementation of *Δexlx–gh5* and Wt. Plants were co-inoculated with a 1:1 mix of Wt & Δ*exlx–gh5.* (Sample sizes, n = 11 per treatment). Y-axis is the log_10_ CFU/g fresh weight and is scaled to the lower limit of detection for the assay (log_10_ CFU/g fresh weight = 2.6). Bars show mean ± SE, and circles are individual biological replicates. **P < 0.005; *ns* non-significant.

To test whether *Δexlx–gh5* has impaired within-host movement and colonization ability, squash seedlings were inoculated with either Wt or *Δexlx–gh5.* At 12 DPI, bacteria were quantified at two sites in each plant: the petiole of the inoculated leaf and the petiole of a second, non-inoculated leaf. At 12 DPI, all of the plants inoculated with the Wt strain were systemically wilting, while plants inoculated with *Δexlx–gh5* were either asymptomatic or had wilt symptoms only in the inoculated leaf (Fig. 6a). At the inoculation site of all plants, the Wt and *Δexlx–gh5* both reached similar cell counts (> 10^9^ CFU/g for Wt, and 10^8^–10^9^ CFU/g for *Δexlx–gh5*) (Fig. 6a). However, in the petiole of a second, non-inoculated leaf Δ*exlx–gh5* only reached cell counts of 10^3^ CFU/g, while the Wt reached 10^9^ CFU/g (Fig. 6a). The Δ*exlx–gh5* strain does not have a growth deficiency in vitro compared to the Wt (Supplemental Figure S1), showing that the attenuation of wilt symptom development and decrease in plant death rates (Figs. 4 and 5) is due to impaired systemic movement of Δ*exlx*–*gh5* and not a difference in intrinsic growth rates. This experiment shows that attenuated virulence in Δ*exlx*–*gh5* mutant (Fig. 4) is related to a decreased ability in systemic xylem colonization^62,63^.

### The *Et–EXLX–GH5* protein functions extracellularly

To test whether the protein products of the *Et–exlx–gh5* locus functions extracellularly (as predicted by the presence of signal peptides for both *Et–exlx* and *Et–gh5*), plants were co-inoculated with a 1:1 mix of Wt and Δ*exlx–gh5.* The aim of this experiment was to assess if the systemic colonization defect of Δ*exlx–gh5* could be rescued by the presence of the expansin and GH5 proteins produced by the Wt strain.

A total of 15 plants were co-inoculated with both Wt and Δ*exlx–gh5.* CFU counts of both strains were quantified at 1 DPI at the inoculation site of four plants (which were then discarded) to ensure both strains were inoculated in equal concentrations, leaving 11 experimental plants (Supplemental Figure S2). At 12 DPI, two of the Wt:Δ*exlx–gh5* co-inoculated plants were asymptomatic, two had symptoms only in the inoculated leaf, and seven had systemic wilt symptoms. At 12 DPI, CFU counts of both the Wt and Δ*exlx–gh5* strains from all of the co-inoculated plants were determined in both the local inoculation site and a petiole of a second, non-inoculated leaf. The average cell count for Wt and Δ*exlx–gh5* in the petiole of a second, non-inoculated leaf both reached similar values of 10^8^ CFU/g (Fig. 6b). This is a notably higher than Δ*exlx–gh5* reaches at the same 12-day time point when inoculated alone (10^3^–10^4^ CFU/g) (Supplemental Figure S3). Three independent experiments (shown in Figs. 4a, 6A and Supplemental Figure S3) resulted in the consistent trend of delayed symptom development in the deletion mutant compared to the Wt strain. The presence of a signal peptide for secretion in both proteins, and the ability of the Wt strain to rescue the systemic colonization defect of Δ*exlx–gh5* together suggests that EXLX and GH5 are secreted and function extracellularly.

The Δ*exlx* and Δ*gh5* single deletion mutants were also co-inoculated in a 1:1 mix to test whether the EXLX and GH5 proteins function extracellularly together as a single complex, or as two independent proteins. The Δ*exlx* deletion mutant is expected to still secrete an intact GH5 protein, and the *Δgh5* deletion mutant is expected to still secrete an intact EXLX protein. If the EXLX and GH5 proteins function independently, the two strains would complement each other in trans. However, this reciprocal complementation was not achieved (Supplemental Figure S4; Supplemental Tables S2 and S3). This result suggests that both proteins need to be produced by the same cell, and that they could function as a single EXLX–GH5 protein complex that assembles before or during secretion. Further assays are needed to confirm that expansin and GH5 are secreted, and whether they assemble into—and function as—a single protein in planta.

### *Erwinia tracheiphila* does not have cellulase or xylanase activity

No expansin protein from any plant, bacteria, fungi, or other microbial eukaryote has detectable enzymatic activity^4,10,16,64^. However, glycoside hydrolases are enzymes that break the glycosidic bond between two or more carbohydrate subunits, and the predominant target of these enzymes is cellulose^48^. It is therefore possible that the *gh5* ORF adjacent to or fused to bacterial expansin genes in some species may confer enzymatic activity. To test whether the *gh5* ORF confers carbohydrate degrading ability to *E. tracheiphila*, the Wt strain (with the intact *exlx–gh5* locus) was evaluated for enzymatic degradation of cellulose and xylan, the two main structural components of plant cell walls and the putative targets of active GH5 enzymes^65^. Neither *E. tracheiphila* culture supernatant nor colonies had detectable hydrolytic activity against cellulose or xylan (Supplemental Figure S5). These results indicate that the function of the GH5 protein is not the enzymatic degradation of plant structural carbohydrates.

### Mutants in flagella and Type IV pili of *Erwinia tracheiphila* display wild type virulence

**Figure 7:**
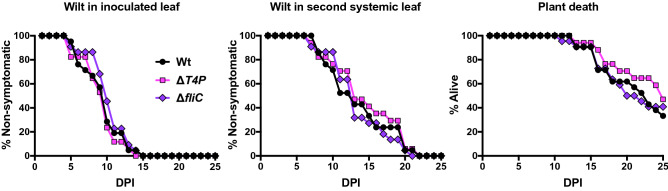
Comparison of virulence between wild type, flagellar deletion mutant (Δ*fliC*) and Type IV Pili deletion mutant (Δ*T4P*). Squash seedlings were inoculated with either Wt, Δ*fliC* or Δ*T4P* strain. Inoculated plants were monitored for first appearance of wilt symptoms in the inoculated leaf, first appearance of systemic wilt symptoms in a second non-inoculated leaf and plant death for 25 days post inoculation (DPI). Summary and statistical analyses are in Tables S4 and S5.

Type IV Pili and flagella are used by some bacterial plant pathogens during systemic movement through xylem^66–69^. To assess whether these cellular components may also contribute to *E. tracheiphila* xylem colonization, deletion mutants were generated for Type IV Pili (Δ*T4P*) and flagella (Δ*fliC*) (Supplemental Table S1). In squash inoculation experiments, there was no difference in the development of wilt symptoms or death rates in groups of plants inoculated with either Δ*T4P*, Δ*fliC*, or Wt (Fig. 7, Supplemental Tables S4 and S5). This indicates that neither Type IV Pili nor flagellar movement contribute to xylem colonization by *E. tracheiphila*, although it is still possible that these loci contribute in other, more subtle, ways to pathogenesis.

## Discussion

Here, we find that the emerging plant pathogen *E. tracheiphila* has horizontally acquired an *exlx–gh5* locus that functions as a virulence factor by conferring the ability to efficiently colonize xylem and cause high rates of plant death. The ability of a pathogen to move systemically through host vasculature—either plant xylem or animal cardiovascular systems—is a high-virulence phenotype, and is associated with development of more severe symptoms than localized infections^70,71^. The ability of a pathogen to reach a high titre and be distributed throughout the host's vasculature is also necessary for vector transmission by providing more opportunities for acquisition^70,72^. In *E. tracheiphila*, bacterial colonization of xylem blocks sap flow, inducing wilt symptoms and causing plant death. The development of systemic wilt symptoms by *E. tracheiphila* also induces a chemical volatile phenotype in symptomatic wild squash (*Cucurbita pepo* ssp. *texana*) that attracts significantly more foraging vectors to wilting leaves^45,73^, and a physical phenotype that facilitates insect vector feeding—and increased pathogen acquisition opportunities—from symptomatic foliage^40,45^. This increase in virulence conferred by the *Et–exlx–gh5* locus induces more severe symptoms in infected plants that both attract obligate insect vectors to infected plants, and facilitates preferential feeding on wilting tissue once they arrive. Together, this suggests that the horizontal acquisition of the *exlx*–*gh5* locus was a key step in the recent emergence of *E. tracheiphila* as a virulent wilt-inducing pathogen that is obligately insect vector transmitted^32,35^.

In ~ 10% of the bacterial species that harbor expansin genes, the expansin is fused to domains of carbohydrate active proteins. The formation of new genes via fusions of multiple modular domains is a key source of evolutionary innovation for organisms across the tree of life^74,75^. A notably high proportion of bacterial species where an expansin co-occurrs with a *gh5* domain are pathogenic to plants, and can move through xylem^7^. This study suggests there may be further emergent properties of the expansin–GH5 protein complex that are uniquely adaptive for xylem-colonizing plant pathogenic bacteria. Healthy plants have effective physical barriers to allow the flow of xylem sap while excluding bacteria. Pit membranes between adjacent tracheids and perforation plates between xylem vessels are openings on a nanometric scale, while most bacteria are ~ 1 µm^76^. One hypothesis is that bacterial expansins may non-enzymatically ‘loosen’ the cellulose and pectin matrix at the perforation plates or at the pit membranes in order to increase their size enough to allow the passage of bacterial cells^77–80^. The ability of *E. tracheiphila* Wt strain to complement Δ*exlx–gh5* in trans supports the working hypothesis that the expansin–GH5 proteins function extracellularly by interacting with xylem structural carbohydrates that would normally prevent bacterial passage. This also suggests that, while the GH5 enzymatic activity has been lost due to truncation of the *Et*–*gh5* gene, the remaining fragment may have been neofunctionalized and is providing an essential (though mechanistically undefined) role in virulence. One possibility is that the GH5 functional domain may physically (but non-enzymatically) interact with plant structural carbohydrates at perforation plates or pit membranes in a way that aids expansin function for loosening of cellulose microfibrils, or vice versa*. *In vitro assays for polysaccharide solubilizing activity (e.g. on isolated vascular bundles) are needed to support a non-enzymatic mechanism for expansin–GH5. It is also possible that microbial expansins and expansin–GH5 constructs may also affect virulence via swarming and attachment processes, as has been found for *Pectobacterium* spp.^21,22^. Further detailed work will be needed to understand the mechanism(s) of action for host interactions and virulence among the diverse microbial species with expansin genes^6,7^.

The phylogenies of the *Et–exlx* and *Et–gh5* ORFs, and their genomic architecture as distinct genes in the same operon, may offer mechanistic insight into how bacterial expansins fuse to carbohydrate active domains. In many Firmicutes, *Pectobacterium* spp. and most *Dickeya* spp. plant pathogens, the *exlx* and *gh5* ORFs are present in the same genome, but are not located directly adjacent to each other in the same operon. Only in *E. tracheiphila, P. stewartii,* and *D. dadantii* is the *exlx* homolog directly adjacent—but not fused to—the *gh5* homolog. This suggests that during a horizontal gene transfer event between an Enterobacteriaceae donor and recipient, an expansin integrated by random chance adjacent to a GH5, and the two ORFs in this operon are now being horizontally transferred together. The assembled protein complex produced by the *exlx–gh5* locus may provide a more efficient mode of action for movement through xylem, promoting the fitness of the host bacteria and providing opportunities for further horizontal transfer of this construct as a single virulence island. From a shared promoter and only ~ 50 nucleotide separation, a fusion of *exlx* and *gh5* into a single ORF is possible from a small deletion mutation. We also note that all three of the bacterial plant pathogens with this construct are agricultural pathogens emerging into intensively cultivated, homogeneous crop plant populations. *Erwinia tracheiphila* has recently emerged into cucurbit agricultural populations^32,35^ and *Pantoea stewartii* infects sweet corn^81^. Both *E. tracheiphila* and *P. stewartii* only occur in temperate Eastern North America—one of the world’s most intensively cultivated regions—despite global distribution of susceptible host plants^82^. *D. dianthicola* causes a virulent wilt disease and is emerging into cultivated potato crops, and is also geographically restricted to Eastern North America and Europe^83–85^. The *exlx–gh5* gene architecture in Enterobacteriaceae, the *gh5–exlx* gene architecture in Xanthomonadaceae and *cbm2–gh5–exlx* gene architecture in *C. michaganensis* (Actinobacteria) is consistent with multiple independent co-occurrences of bacterial expansins with evolutionarily distinct *gh5* domains, and may be an example of functional convergence.

There is constant risk that agro-ecosystems will be invaded by virulent microorganisms, and the increasing homogeneity in crop plant populations may select for novel pathogens with non-canonical virulence mechanisms. The recent realization that microbial expansin genes are present in phylogenetically diverse xylem-colonizing bacterial and fungal species—including almost all of the most economically damaging bacterial and fungal wilt pathogens – and the function of expansins to increase *E. tracheiphila* virulence suggest these genes may be an under-appreciated virulence factor. The emergence of virulent plant pathogens that systemically colonize xylem is especially alarming because plants do not have inherent genetic resistance against xylem-dwelling vascular pathogens^86^. The increase in *E. tracheiphila* virulence conferred by expansin, the presence of this gene in many other bacterial and fungal wilt-inducing plant pathogen species, and the amenability of microbial expansins to horizontal gene transfer suggest this gene may be an under-appreciated virulence factor in taxonomically diverse agricultural pathogens.

## Methods

### Bacterial strains, culture media and plant cultivation

All bacterial strains used in this study are listed in Supplemental Table S1. Throughout this work, we used a rifampicin resistant variant of *E. tracheiphila* BHKYR (Wt)^32^. *Escherichia coli* TOP10 and PIR1 strains for used for routine cloning, and the *E. coli* strain S17-1λ was used as the donor for conjugation. *E. tracheiphila* was grown in KB liquid media or agar at room temperature (RT), and *E. coli* strains in LB media or agar at 37 °C, unless otherwise specified. Antibiotics were added to liquid or agar media at the following concentrations: rifampicin, 50 μg/ml; ampicillin or carbenicillin, 100 μg/ml; chloramphenicol 5 μg/ml; kanamycin 50 μg/ml. All in planta experiments were conducted with organic ‘Dixie’ variety crookneck squash bought from Johnny’s Seeds (https://www.johnnyseeds.com/). Plants were grown in potting mix in standard six cell seedling trays in a greenhouse environment set to 25 °C, 70% humidity, and a 12 h day:12 h night light cycle.

### Visualization of fluorescent *Erwinia tracheiphila* in wilting squash seedlings

*Erwinia tracheiphila* BuffGH was transformed with a plasmid carrying the mCherry gene for visualization of fluorescent cells in symptomatic squash seedlings. Competent *E. tracheiphila* were prepared as described previously^32,87^. Briefly, cells were prepared by growing *E. tracheiphila* to an OD_600_ of 0.02. Cells were then washed with decreasing volumes, once with chilled sterile Milli-Q water and twice with 10% glycerol, and resuspended in 1/100 volume of chilled 10% glycerol. Plasmid pMP7605 was used for electroporation in a 0.2-cm cuvette, at 2.5 kV for 5.2–5.8 ms. Cells were incubated at room temperature without shaking for 1 h in 3 ml KB liquid and then plated in KB agar with ampicillin. Colonies of fluorescent *E. tracheiphila Et* (pMP605) were obtained after 5 days at room temperature. Ten µl of a *Et* (pMP7605) stationary culture were used for inoculating 2 week-old squash seedlings (at the two leaf stage), and confocal microscopic observations were performed once symptoms appear using fresh longitudinal cuts of the inoculated petiole.

### Phylogenetic reconstruction of the *exlx* and *gh5* genes and comparison of domain architecture

The amino acid sequences of the expansin (WP_046372116.1) and *gh5* (WP_016193008.1) ORFs in the *E. tracheiphila* reference strain^29^ were used as queries to identify expansin and *gh5* homologs using the BLASTP web interface^88^. A taxonomically representative sample of the top BLASTP hits for each gene were aligned using MAFFT v. 7.305b and default parameters^89^. The expansin alignment was trimmed visually such that the two canonical expansin domains were conserved in the alignment, and the *gh5* alignment was trimmed with trimAI using the—automated 1 option^90^. For both alignments ProtTest v. 3.4.2 was used to identify the best-fitting substitution model by BIC score, which was WAG + G for the expansin gene alignment and LG + I + G for the GH5 alignment^91^. The GyrB species tree was constructed by using the *E. tracheiphila* GyrB sequence (KKF36621.1) as a query on the BLASTP web interface^88^. The GyrB amino acid sequences from species known to have an expansin gene or an expansin fusion to a domain from a carbohydrate active protein were downloaded and added to a multi-FASTA (https://github.com/lshapiro31/gh5.expansin.phylogenetics). The GyrB sequences were aligned with MAFFT v. 7.305b and default parameters^89^.

Phylogenetic trees were reconstructed using maximum likelihood with RAxML^92^ and the appropriate evolutionary model on the CIPRES server^93^. The expansin tree was reconstructed with 1000 bootstrap pseudoreplicates, and the GH5 and GyrB trees were reconstructed with 100 bootstrap pseudoreplicates. The bootstrapped pseudosamples were summarized with SumTrees v. 4.4.0^94^. The resulting phylogeny was visualized in the R statistical environment using the ggtree library^95,96^. Amino acid sequences were analyzed with NCBI CBD tool to identify domain architecture^47^, and signal peptides were predicted with SignalP^50^. The genomic context of the *Et–exlx–gh5* locus was visualized with genoPlotR^97^. Alignment files and phylogenetic scripts are available at https://github.com/lshapiro31/gh5.expansin.phylogenetics.

### Construction of deletion mutants

Mutants with a deletion in the *exlx–gh5* operon, *exlx* gene, *gh5* gene, the Type IV pili operon and the *fli*C gene were generated from an *E. tracheiphila* isolate BHKYR parental strain by double homologous recombination, using the suicide plasmid pDS132^98^. This plasmid was improved by inserting in the *Xba*I site, a constitutive *mCherry* gene amplified from plasmid pMP7605^99^ using primers JR72 and JR73 (Supplemental Table S6). The resulting plasmid (pJR74, Supplemental Table S1) allows rapid screening of conjugants colonies and colonies that have lost the plasmid. For the target genomic region to create each mutant, regions upstream of the target locus were amplified with primers pair F5 and R5, and downstream regions were amplified with primer pair F3 and R3 (See Supplemental Table S6 for specific primer names and sequences). An ampicillin resistance *bla* gene, coding for Beta-lactamase was amplified from pDK46^87^ using primers LS23 and LS24. Constructions consisting on each upstream and downstream region flanking the *bla* gene were used for *exlx–gh5*, *gh5*, *fliC* and Type 4 Pili mutants, while a construction with no flanked antibiotic cassette was prepared for the *exlx* deletion. All constructions were assembled using the Gibson Assembly Master Mix (New England Biolabs, Ipswich, MA), and then each was reamplified with nested primers containing *Sac*I restriction site (primers *Sac*I-F and *Sac*I-R, Supplemental Table S6). Constructions for *exlx–gh5*, *exlx*, *gh5*, *fliC* and Type 4 Pili deletion were inserted into the *Sac*I site of plasmid pJR74, obtaining plasmids pJR150, pJR323, pJR324, pJR74a and pJR149, respectively (Supplemental Table S1). These plasmids were transformed into *Ec*-PIR1 for preservation, and then into *Ec*-S17 for conjugation using *E. tracheiphila* as recipient. MCherry fluorescent *E. tracheiphila* conjugants were obtained in KB agar with rifampicin and chloramphenicol, then a few colonies were picked and grown in 3 ml liquid KB with chloramphenicol to stationary phase, and 100 µl were spread in KB agar with 5% sucrose and carbenicillin (or no antibiotic in the case of *exlx* deletion). Non-fluorescent, chloramphenicol-sensitive colonies were picked, PCR checked for the correct deletion and cryogenically stored in 15% glycerol at − 80 °C.

### Genetic complementation

A new integration plasmid, specific for a neutral region in the chromosome of *Et*-BHKYR, was constructed from plasmid pJR74. To create this plasmid, two ≈ 0.8 Kb adjacent DNA fragments were PCR amplified form *Et*-BHKYR genomic DNA using primer pairs JR143–JR144, and JR145–JR146 (Supplemental Table S6). These fragments were ligated using the Gibson Assembly Master Mix (New England Biolabs, Ipswich MA), and reamplified using primers JR143 and JR146. The ≈ 1.6 Kb product was inserted in the SacI site of pJR74, generating plasmid pJR315 (Supplemental Table S1). Single cutting *Xho*I and *Bgl*II sites were engineered in the middle of the amplified neutral regions, which can be used for the insertion of complementation genes. For the complementation of the *exlx–gh5* locus or the individual *exlx* gene, the genomic region together with its natural promoter were amplified from *E. tracheiphila* genomic DNA using primers JR152 and JR154, or JR152 and JR153 (Supplemental Table S6) respectively, and DNA products were inserted into the *Xho*I site of pJR315. Promoter regions for expression of the *exlx–gh5* operon have not been characterized, but expression of individual ORFs in an operon is often directed from an upstream shared promoter. It is therefore reasonable to assume expression of the *gh5* ORF is directed from a shared promoter region upstream of *exlx*^100^*.* For this reason, the single Δ*gh5* mutant was complemented with the full operon (*exlx–eng*) to include the promoter region of *exlx*. Each resulting plasmid was transformed into *Ec*-S17-1λ cells, which were then used as donors for conjugation with mutant strains Δ*exlx–gh5,* Δ*exlx* or Δ*gh5* as recipients. Conjugant colonies were used for negative selection with sucrose, as described above, and colonies carrying the *exlx–gh5* operon or the *exlx* gene integrated in the expected site were confirmed by PCR.

### In planta inoculation experiments

In planta virulence assays were performed by inoculating squash seedlings with *E. tracheiphila* Wt and derived strains, and monitoring wilt symptom development for approximately three weeks (between 21 and 25 days per experiment). To create inoculum, one bacterial colony of each strain was picked and added to 3 ml of liquid KB media with the appropriate antibiotic, and grown with shaking for 24 h. Then, 2–3-week-old squash seedlings (2–3 true leaves) were inoculated by manually inducing a wound where xylem was exposed in the petiole at the base of the first true leaf and adding 10 μl of culture containing ≈ 1 × 10^7^ bacterial cells directly into the wound. In each experiment, an equal number of plants were inoculated in each group; however, plants that were inadvertently damaged during watering or symptom assessment were removed from experiments. Plants were kept at 25 °C, 70% humidity, and a 12 h day:12 h night light cycle 25 °C, and monitored daily for appearance of first symptoms in the inoculated leaf, appearance of wilt symptoms in a second non-inoculated leaf, and plant death.

### In planta colony forming unit (CFU) counts

Bacterial colony forming units (CFU) counts were determined from plants inoculated with *Et-*BHKY Wt and derived strains. Bacterial cells can be obtained directly from petioles of infected plants (Fig. 1). Two cm samples of the petiole from the inoculated leaf, or from a second non-inoculated leaf were cut from the plants and washed briefly with 70% ethanol (EtOH). Excess EtOH was removed with a paper towel and petioles were surface-sterilized over a gas flame for 1 s and placed in a sterile plastic petri dish. From each petiole sample, 10–15 disks small disks (< 1 mm) were manually cut with a sterile blade and collected in a 2 ml microtube. The weight of each 2 ml tube with all leaf disks was recorded to be used for normalizing CFU per gram of plant tissue in each sample, and 500 μl of chilled PBS was added to each tube. After an incubation of 40 min on ice (vortexing every 10 min) 200 μl of PBS from each tube was pipetted into a new microtube and used for serial dilutions and plating onto KB agar with rifampicin. Bacterial CFU per gram of fresh tissue was calculated.

For obtaining CFUs of individual strains in plants co-inoculated with *E. tracheiphila* Wt and Δ*exlx–gh5* mutant, serial dilutions were plated in both KB with rifampicin and KB with rifampicin and carbenicillin agar plates. CFU of carbenicillin resistant colonies represent the Δ*exlx–gh5* strain. CFUs of Wt was determined as the count of total CFUs—carbenicillin resistant CFUs.

### Statistics

Statistical analyses were performed using Prism version 7.0 (GraphPad Software, La Jolla California USA, www.graphpad.com). Curves following initial symptoms in first leaf, systemic wilt in second leaf, and plant death from each experiment were compared using the built-in Log-rank (Mantel–Cox) test for survival analysis. In the cases where significant differences were found (*p* < 0.05), pairwise comparisons were tested using the same analysis^101^. For comparisons of bacterial CFU in planta, CFU data and its log_10_-transformed values were checked for Gaussian distribution using the Shapiro–Wilk normality test. Since neither CFU data distribution nor the transformed Log_10_ values distribution passed the normality test, the Kruskal–Wallis non-parametric was used test to analyze if the medians vary significantly among experimental groups (*p* < 0.05). In the cases where differences were found, Dunn's multiple comparisons test was used to test for pairwise differences between groups.

### Testing for cellulase and xylanase activity

Cellulase activity from cell-free supernatants of Wt and Δ*exlx–gh5* cultures were tested for extracellular enzymatic activity against cellulose. The Wt and Δ*exlx–gh5* strains were grown in 10 ml of liquid KB media for 48 H. Cultures were centrifuged at 7000 rpm for 10 min, and each supernatant was filter-sterilized. Supernatants and 1 mg/ml cellulase (Sigma), were spotted in 1% agar, 1% Carboxy Methyl Cellulose (CMC) plates. Plates were then incubated at 30 °C for 48 h, and flooded with Gram’s Iodine. Halos were imaged after 24 h, at RT.

A colony of *E. tracheiphila* grown on KB agar plates was used to test for extracellular xylanase activity. A xylanase producing strain of *Streptomyces lividens* was used as a positive control. Bacterial culture from each species was spotted on the surface of a KB agar plate, and grown at RT for 4 days. An overlay of 1% agar and 1% xylan was spread on top of the grown colonies, and plates were incubated at 30 °C for 48 h. Plates were flooded with 1% congo red and incubated for 10 min before discarding the Congo red solution. Plates were then flooded with 1 N NaOH, and incubated for 10 min. NaOH was discarded and plates were imaged after 24 h at room temperature.

## Supplementary information

Supplementary Information.
