# Supplementary material for: A horizontally acquired expansin gene increases virulence of the emerging plant pathogen Erwinia tracheiphila
Source: Sci Rep. 2020 Dec 10;10:21743. doi: 10.1038/s41598-020-78157-w (PMC7729394; doi:10.1038/s41598-020-78157-w)
Supplement: Supplementary file 1 — Supplementary Information. [file 41598_2020_78157_MOESM1_ESM.pdf]

Supplemental material

A horizontally acquired expansin gene increases virulence of the emerging plant pathogen *Erwinia tracheiphila*

Jorge Rocha, Lori R. Shapiro, Roberto Kolter

Running title*:* *An expansin increases* Erwinia tracheiphila *virulence*

Data Deposition Statement: Analysis scripts and input files associated with reconstruction of phylogenetic trees are available at https://github.com/lshapiro31/gh5.expansin.phylogenetics

Supplemental Tables

Supplemental Table S1. Strains and plasmids used in this study.

Strains	Description	Reference
Erwinia tracheiphila BHKY	*Erwinia tracheiphila* parental Wild type strain. Spontaneous rifampicin resistant. Parental strain for mutants and complemented variants.	Rojas, E. Saalau, et al. *Phytopathology* 103.9 (2013): 900-905. https://doi.org/10.1094/PHYTO-11-12-0301-R
Erwinia tracheiphila BuffGH	Erwinia tracheiphila Wildtype isolate, used for fluorescence visualization of bacterial cells during infection.	28
Δ*exlx-gh5*	Deletion mutant strain; *exlx-gh5:bla* genetic exchange. Ampicillin resistant.	This study
Δ*exlx*	Deletion mutant strain; clean deletion in N terminal coding region of *exlx*.	This study
Δ*eng*	Deletion mutant strain; *gh5:bla* genetic exchange. Ampicillin resistant.	This study
Δ*fliC*	Deletion mutant strain; *fliC*:*bla* genetic exchange. Ampicillin resistant.	This study
ΔT4P	Deletion mutant strain; *T4P*:*bla* genetic exchange. Ampicillin resistant.	This study
Δ*exlx-gh5*(cEXLX-GH5)	Mutant strain Δ*exlx-gh5,* complemented with wildtype promoter and *exp-gh5* coding region, inserted in a neutral chromosomic region.	This study
Δ*exlx*(EXLX)	Mutant strain Δ*exlx,* complemented with wildtype promoter and *exlx* coding region, inserted in a neutral chromosomic region.	This study
Δ*eng*(EXLX-ENG)	Mutant strain Δ*egh5,* complemented with wildtype promoter and *exlx-gh5* coding region, inserted in a neutral chromosomic region.	This study
E. coli Top10	*E. coli* strain used for cloning.	
E. coli PIR1	*E. coli* srtain for expression of R6K replication origin.	
E. coli S17-1λ	*E coli* strain used as donor for conjugation.	
Plasmids		
pDS132	Suicide plasmid for allelic replacement.	91
pMP7605	Template for the amplification of the *mcherry* gene	92
pJR74	Derived from pDS132, *mcherry* gene inserted in the *Xba*I site.	This study
pKD46	Template for *bla* gene (ampicillin resistance cassete).	80

pJR315	Integration plasmid for *E. tracheiphila*.	This study
pJR150	Plasmid carrying the construction for gene exchange between the *exlx-gh5* operon and the bla gene, derived from pJR74.	This study
pJR323	Plasmid carrying the construction for *exlx* deletion, derived from pJR74.	This study
pJR324	Plasmid carrying the construction for *gh5::bla* gene exchange, derived from pJR74.	This study
pJR74a	Plasmid carrying the construction for *fliC:bla* gene exchange, derived from pJR74.	This study
pJR149	Plasmid carrying the construction for Type 4 Pili operon gene exchange with *bla* gene, derived from pJR74	This study
pJR358	Plasmid carrying the construction for *exlx-eng* operon integration into the chromosome of Et-BHKY, derived from pJR315.	This study
pJR357	Plasmid carrying the construction for *exlx* gene integration into the chromosome of Et-BHKY, derived from pJR315.	This study

Supplemental Table S2. Summary of *in planta* inoculation experiment comparing virulence traits between mutant strains Δ*exlx*, Δ*gh5* and a 1:1 mix of both Δ*exlx* and Δ*gh5* mutants, (Supplementary Figure S4).

	Average number of days until:			Number of plants with symptoms at end of experiment (day 23)			
**Strain**	**Local wilt symptoms (first leaf)**	**Systemic wilt symptoms (second leaf)**	**Death**	**Local wilt symptoms (first leaf)**	**Systemic wilt symptoms (second leaf)**	**Died**	**Total plants**
**Δ*exlx***	9.33	16.25	17.33	15 (83%)	8 (44%)	3 (16%)	18
**Δ*gh5***	11.69	16.88	17.25	16 (88%)	8 (44%)	4 (22%)	18
**Δ*exlx* and Δ*gh5* mix**	11.11	14.76	19.05	15 (83%)	8 (44%)	4 (22%)	18

Supplemental Table S3. Log-rank (Mantel-Cox) tests for assessing statistical differences in virulence experiments from Supplemental Figure S4, comparing Δ*exlx*, Δ*gh5* and Δ*exlx*:*Δgh5* mix.

Compared treatment groups	First leaf symptoms		Second systemic leaf symptoms		Death of plants	
	Chi square	*p* value	Chi square	*p* value	Chi square	*p* value
All groups	0.5335	0.7659	0.05808	0.9714	0.2023	0.9038

Supplemental Table S4. Summary of *in planta* inoculation experiment comparing virulence traits between strains Wt, Δ*fliC* mutant and Δ*T4P* mutant, corresponding to Figure 7.

	Average number of days until:			Number of plants with symptoms at end of experiment (day 23)			
**Strain**	**Local wilt symptoms (first leaf)**	**Systemic wilt symptoms (second leaf)**	**Death**	**Local wilt symptoms (first leaf)**	**Systemic wilt symptoms (second leaf)**	**Died**	**Total plants**
**Wt**	9.43	13.62	18.86	21 (100%)	21 (100%)	14 (66%)	21
**Δ*fliC***	10.23	13.45	17.54	22 (100%)	22 (100%)	13 (59%)	22
**Δ*T4P***	9.18	14.53	19.67	17 (100%)	17 (100%)	9 (52%)	17

*22 plants were inoculated in each group; several plants were removed from the analysis due to physical damage.

Supplemental Table S5. Log-rank (Mantel-Cox) tests for assessing statistical differences in virulence experiments from figure 7, comparing Wt, Δ*fliC* and Δ*T4P*.

Compared treatment groups	First leaf symptoms		Second systemic leaf symptoms		Death of plants	
	Chi square	*p* value	Chi square	*p* value	Chi square	*p* value
All groups	1.623	0.6543	1.324	0.7235	2.156	0.5406

Supplemental Table S6. Oligonucleotides used in this study.

Primer Name	Description and sequence (5' to 3')	Modification
	***bla* cassette amplification**	
LS23	acttttcggggaaatgtgc	
LS24	acgttaagggattttggtca	
	***mcherry* gene amplification**	
JR72	tcttctagacgttttcttacttgtacagctc	*Xba*I site
JR73	tcttctagaaattcttgacaattaatcatcg	*Xba*I site
	***exlx-gh5*::*bla* exchange**	
**LS52 (Fwd 5')**	acccgttaatgcaccagaac	
**LS53 (reamplification)**	gaggagctcttatttcgatgatggttttatgg	*Sac*I site
**LS54 (Rev 5')**	gcacatttccccgaaaagtagttaaaacagcgcagatgg	LS23 tag
**LS55 (Fwd 3')**	tgaccaaaatcccttaacgtacagaggatgccctggtaag	LS24 tag
**LS57 (reamplification)**	gaggagctccgcaaatcatcaccagtcag	*Sac*I site
**LS56 (Rev 3')**	gaggtatatcccggcctgac	
	***exlx* clean deletion**	
**JR175 (Fwd 5')**	Cagaactgacgcttacctcc	
**JR176 (reamplification)**	GAGGAGCTCtcgcttctttatagagctgc	*Sac*I site
JR194 (Rev 5')	gttgagctggtaaggactaagg gaaagtcagagccgctattG	JR195 tag
JR195 (Fwd 3')	Caatagcggctctgactttcccttagtccttaccagctcaac	JR194 tag
**JR179 (reamplification)**	GAGGAGCTCatcgataacgctatccacac	*Sac*I site
**JR180 (Rev 3')**	Gtatttcgtagccaatctctg	
	***gh5::bla* exchange**	
**JR181 (Fwd 5')**	Gaatcaggcagacttggttc	
**JR182 (reamplification)**	GAGGAGCTCgtgattacaaccatgctttcg	*Sac*I site
**JR183 (Rev 5')**	GCACATTTCCCCGAAAAGT gcatgctgctacagataacc	LS23 tag
**JR184 (Fwd 3')**	TGACCAAAATCCCTTAACGT gataaagcagaaggagcatc	LS24 tag
**JR185 (reamplification)**	GAGGAGCTCcccactgatgttatcgctc	*Sac*I site
**JR186 (Rev 3')**	Catgctgtttttttatattacctgc	
	**Integration plasmid**	
**JR143 (Fwd 5')**	GAGGAGCTCcttcaaaaatacgttcacacc	*Sac*I site
**JR144 (Rev 5')**	gttcatacatggtcaaacctg AGATCTCTCGAG catttatccgtgctgatctg	*Xho*I, *Bgl*II sites, JR145 tag
**JR145 (Fwd 3')**	cagatcagcacggataaatg CTCGAGAGATCT caggtttgaccatgtatgaac	*Xho*I, *Bgl*II sites, JR144 tag
**JR146 (Rev 3')**	GAGGAGCTCaataccaatcaggaccacac	*Sac*I site
	**Complementation of *exlx-gh5* and *exlx***	

**JR152 (Fwd)**	CTCCTCGAGggaagattttcatcagcacc	*Xho*I site
**JR153 (Rev *exlx* only)**	CTCCTCGAGctgtcatgtcctgtattatatattgtg	*Xho*I site
**JR154 (Rev *exlx-eng*)**	CTCCTCGAGgacagtaccagtatcctgacg	*Xho*I site
	***gh5::bla* exchange**	
**LS1 (Fwd 5')**	CTGGCGATAGTGGTGGAG	
**JR62 (reamplification)**	GAGGAGCTCagtcagcgaaatcttcacg	*Sac*I site
**LS2 (Rev 3')**	GCACATTTCCCCGAAAAGTgtgccatgataaagtttcctg	LS23 tag
**LS3 (Fwd 3')**	TGACCAAAATCCCTTAACGTctgctgcagggctaataac	LS24 tag
**JR63 (reamplification)**	GAGGAGCTCggaatacgtagcagatcatcg	*Sac*I site
**LS4 (Rev 3')**	cgacaaagcggataacactc	
	***T4P::bla* exchange**	
**LS46 (Fwd 5')**	ctgcggaaaatgaccttacc	
**LS48 (reamplification)**	GAGGAGCTCacggtcggtggaagaagtc	*Sac*I site
**LS47 (Rev 5')**	GCACATTTCCCCGAAAAGTtcaatcagggtaaatccttgc	LS23 tag
**LS49 (Fwd 3')**	TGACCAAAATCCCTTAACGTctaccctggtggtagcgatg	LS24 tag
**LS51 (reamplification)**	GAGGAGCTCgatgggatggtcaggtgtg	*Sac*I site
**LS50 (Rev 3')**	atgtattaatccgcccgaag	

Supplemental Figures and Legends

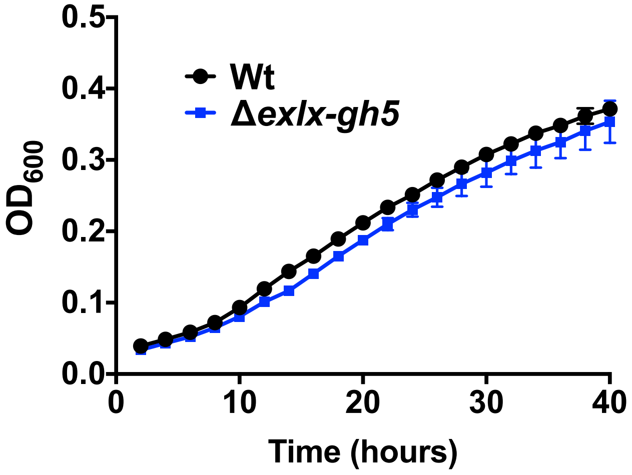

Supplemental Figure S1. *In vitro* growth of Wt and Δ*exlx-gh5*. A single colony of Wt or Δ*exlx-gh5* strains were picked into 3 ml of liquid KB media and grown for 48 h at 25°C with shaking. Then, 1 ml of each culture was washed once with 1 volume of PBS and diluted with fresh KB media to OD_{600} of 0.05. Four replicates of 300 µl were placed in a clear 96-well microplate and growth was followed in these standing liquid cultures using a microplate reader, by measuring absorbance at 600 nm every 2 h for 40 h at a constant temperature of 25°C. Average ± SD is shown.

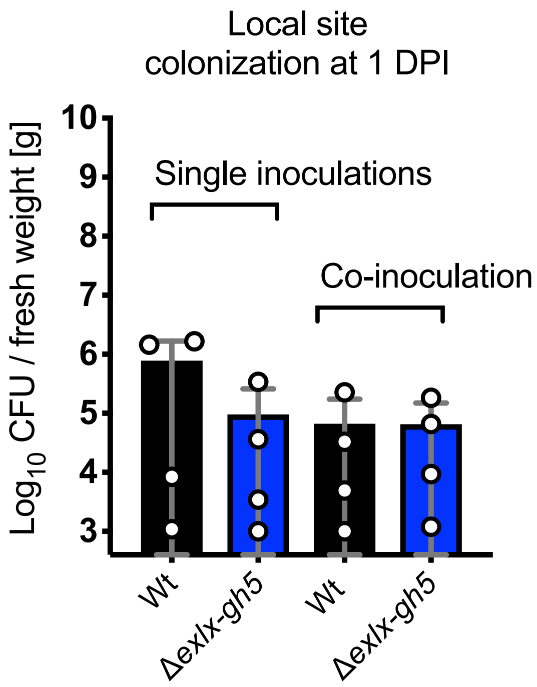

Supplemental Figure S2. Colonization of Wt and Δ*exlx-gh5* mutant in single inoculations and co-inoculation. CFU were counted at 1-day post inoculation (DPI) from the local inoculation site of plants inoculated with Wt (n=4), *Δexlx-gh5* (n=4), and co-inocualted with Wt and *Δexlx-gh5* (n=4). Y-axis is the $\log_{10}$ CFU/gram fresh weight and is scaled to the lower limit of detection for the assay ($\log_{10}$CFU/gram fresh weight = 2.6). Bars show mean ± SE, and circles are individual biological replicates.

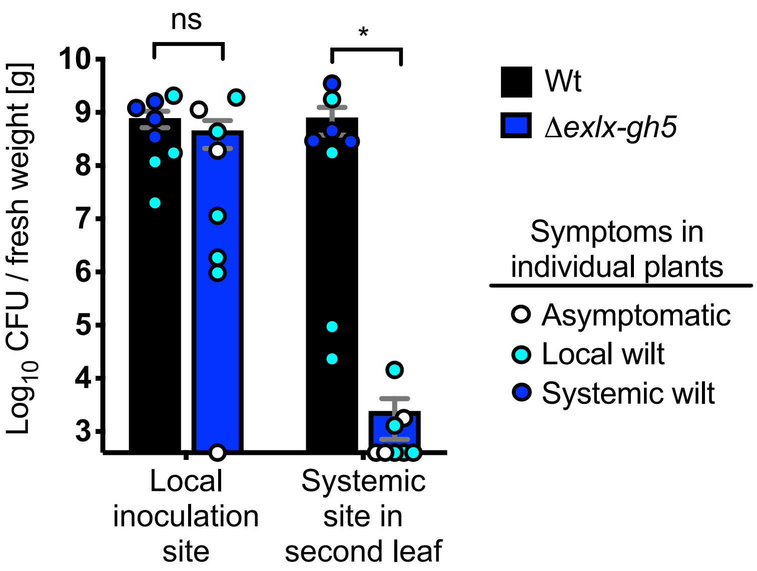

Supplemental Figure S3. Single inoculation controls for the co-inoculation experiment in figure 6B. Plants inoculated with Wt or mutant strain were sampled at 12 DPI (Sample sizes, n=8 per treatment). Y-axis is the $\log_{10}$ CFU/gram fresh weight and is scaled to the lower limit of detection for the assay ($\log_{10}$CFU/gram fresh weight = 2.6). Bars show mean ± SE, and circles are individual biological replicates. * $P < 0.05$; ns, non-significant.

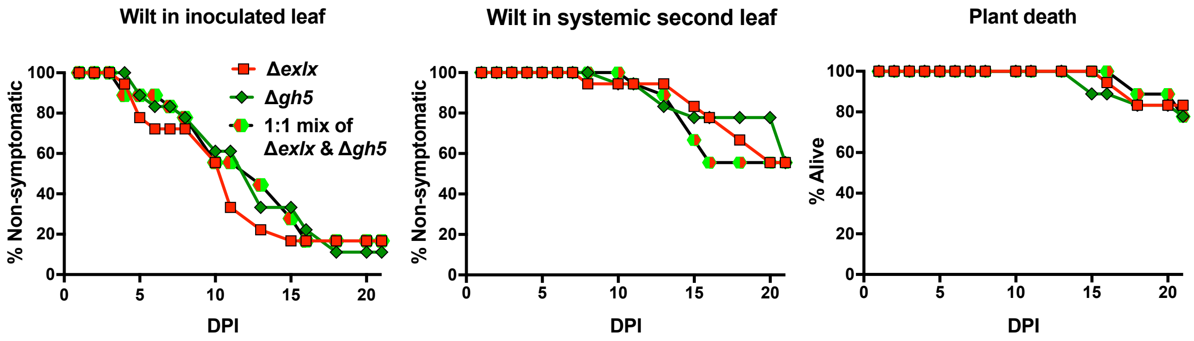

Supplemental Figure S4. *In trans* complementation of Δ*exlx* and Δ*gh5*. Plants were co-inoculated with both the Δ*exlx* and Δ*gh5* deletion mutants. Inoculated plants were monitored for first appearance of wilt symptoms in the inoculated leaf, first appearance of systemic wilt symptoms in a second leaf and plant death for 21 days post inoculation (DPI). Summary and statistical analyses are in Tables S2 and S3.

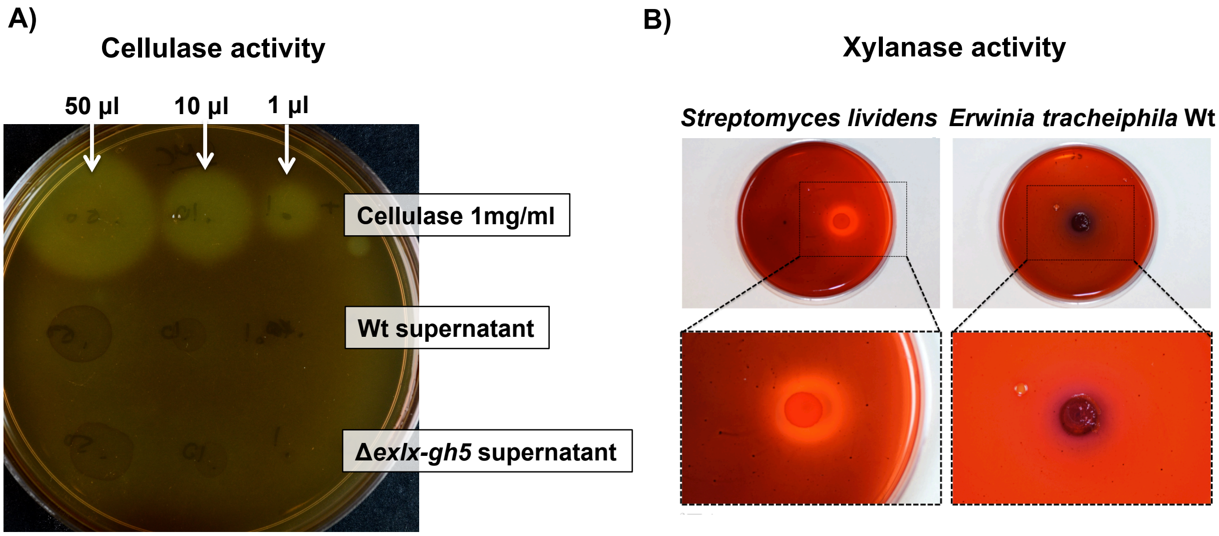

Supplemental Figure S5. **Tests to detect *E. tracheiphila* cellulase and xylanase activity *in vitro*. A)** Commercial cellulase and supernatants of Wt and the Δ*exlx-gh5* mutant were spotted in media containing agar and 1% CMC, incubated at 30°C for 48 h, and then flooded with Gram's Iodine. Halos were imaged after 24 h at RT. **B)** *E. tracheiphila* and the xylan degrading species *Streptomyces lividens* were grown in KB agar. An overlay of 1% xylan and 1% agar was spread on top of the colonies. Plates were incubated at 30°C for 48 h, and flooded with 1% Congo Red. Halos were imaged and measured after 24 h at room temperature.
